# Enhancement of simeprevir and sofosbuvir anti-ZIKV activity by iNOS-derived NO *in vitro*

**DOI:** 10.1590/0074-02760250315

**Published:** 2026-09-28

**Authors:** Rômulo José Soares-Bezerra, Karen dos Santos Charret, João Marcos Beserra Rocha da Silva, Joana Miranda Mamprim, Lucas de Souza Leite, Richard de Almeida Lima, Daniela del Rosário Flores Rodrigues, Alexandre dos Santos da Silva, Natiele Carla da Silva Ferreira, Marcelo Alves Pinto

**Affiliations:** 1 Fundação Oswaldo Cruz-Fiocruz, Instituto Oswaldo Cruz, Laboratório de Desenvolvimento Tecnológico em Virologia, Rio de Janeiro, RJ, Brasil; 2 Fundação Oswaldo Cruz-Fiocruz, Instituto Oswaldo Cruz, Laboratório de Bioquímica de Tripanossomatídeos, Rio de Janeiro, RJ, Brasil; 3 Fundação Oswaldo Cruz-Fiocruz, Instituto Oswaldo Cruz, Laboratório de Comunicação Celular, Rio de Janeiro, RJ, Brasil; 4 Universidade Federal do Rio de Janeiro, Instituto de Ciências Biomédicas, Rio de Janeiro, RJ, Brasil

**Keywords:** ZIKV, simeprevir, sofosbuvir, iNOS

## Abstract

**BACKGROUND:**

Zika virus (ZIKV) was first identified in the Americas in 2015, with Brazil experiencing a major epidemic marked by teratogenic effects during early pregnancy. *Ex vivo* studies of microglia from fatal ZIKV-induced microcephaly cases revealed elevated levels of inducible nitric oxide synthase (iNOS) and nitric oxide (NO). Direct-acting antivirals (DAAs) have been proposed as potential ZIKV treatments.

**OBJECTIVES:**

This study aimed to investigate the effects of two DAAs, simeprevir and sofosbuvir, on iNOS expression and NO production during *in vitro* ZIKV infection.

**METHODS:**

Molecular docking analyses were performed to assess the interaction between DAAs and the iNOS enzyme. ZIKV-infected Vero CCL-81 cells were treated with simeprevir or sofosbuvir (10 µmol/L), and iNOS expression, NO production, and cytotoxicity were evaluated.

**FINDINGS:**

Both DAAs demonstrated strong binding affinity to the iNOS active site. Compared with the untreated infected controls, treatment with simeprevir and sofosbuvir significantly upregulated iNOS expression and increased NO production by approximately 60%. No cytotoxic effects were observed under the experimental conditions.

**MAIN CONCLUSIONS:**

Simeprevir and sofosbuvir not only exhibit antiviral properties but also may enhance the host antiviral response via modulation of the NO pathway, suggesting a dual mechanism of action against ZIKV.

Zika virus (ZIKV) is a member of the *Flaviviridae* virus family, which comprises a large group of enveloped viruses with a single-stranded RNA genome of positive polarity.^1^ ZIKV is spherical and approximately 40 nm in diameter; its genome is composed of nonsegmented positive-sense, single-stranded RNA, which has a single open reading frame (ORF) of approximately 10,794 nucleotides encoding 3,419 amino acids. The main ZIKV proteins include seven nonstructural proteins (NS1, NS2A, NS2B, NS3, NS4A, NS4B, and NS5) and three structural proteins (C, prM, and E, which encode the capsid, membrane, and viral envelope, respectively).^2^ ^,^ ^3^ ^,^ ^4^

ZIKV causes an arboviral disease that is transmitted mainly by mosquitoes of the *Aedes* genus.^5^ It was isolated for the first time in 1947 in a blood sample from a *rhesus* monkey during a study on yellow fever carried out in the Zika forest in Uganda.^6^ In the following year, in the same region, the virus was isolated from samples of *Aedes africanus* mosquitoes; nevertheless, in 1948, a serological study was conducted in the same locality, and the seroprevalence of anti-Zika antibodies was 6.1% among residents of regions close to Uganda.^7^ The relationship between ZIKV as a disease-causing agent in humans occurred in 1953 in Nigeria.^8^

The first detection of ZIKV in the Americas occurred in early 2015 in Brazil, indicating the occurrence of autochthonous transmission of ZIKV in the western hemisphere.^9^ However, ZIKV was introduced in Brazil before 2015, between October 2012 and May 2013, and remained undetectable until 2015.^10^ During the outbreak of ZIKV in Brazil, the main symptoms presented by patients included fever, retro-orbital pain, myalgia, arthralgia, headache, maculopapular and pruritic eruptions, oedema of extremities, nonpurulent conjunctivitis, and digestive disorders.^9^

Before the ZIKV outbreak in Brazil in 2015, approximately 200 cases of microcephaly were registered annually in Brazil; between the second half of 2015 and January 2016, more than 4,000 suspected cases of microcephaly were registered, although a possible diagnosis can be considered incorrect or excessive in some cases.^4^

The detection of viral RNA in the amniotic fluid of pregnant women represented the first diagnosis of intrauterine transmission of ZIKV.^11^ ZIKV RNA was subsequently detected in the tissues of foetuses infected during pregnancy and in the brain tissues of stillborn foetuses with microcephaly.^12^ ^,^ ^13^ However, ZIKV neurotropism was demonstrated in 1952.^6^

To date, no specific approved treatments or vaccines against ZIKV have been available. Although several vaccine candidates, including inactivated, live-attenuated, DNA, mRNA, and virus-vectored platforms, have advanced to clinical studies (phases I and II), the global decline in epidemic cases after 2017 has rendered phase 3 efficacy trials potentially infeasible. Nevertheless, the risk of ZIKV re-emergence and the occurrence of another major epidemic in the future cannot be excluded.^14^ ^,^ ^15^ Consequently, exploring potential therapeutic targets for ZIKV may provide viable treatment options.

Recent data have demonstrated a correlation of ZIKV infection and microcephaly with the activation of the inflammasome complex and increased expression of inducible nitric oxide synthase (iNOS).^16^ ^,^ ^17^ iNOS is an isoform of the heme protein NOS, which catalyses the conversion of L-arginine to citrulline, generating a free radical, nitric oxide (NO). iNOS is expressed mainly in macrophages as part of antiviral pathways,^18^ inducing high levels of NO,^19^ an innate mechanism that is also involved in the killing of invasive tumour cells and parasites, beta cell injury in autoimmune diabetes, and a key role in immunopathological phenomena of many inflammatory diseases.^20^ On the other hand, many research groups worldwide focus on screening new molecules or repositioning drugs and searching for effective treatments for ZIKV infection, including DAAs currently used in chronic hepatitis C virus (HCV) treatment.^16^ ^,^ ^17^ ^,^ ^21^ ^,^ ^22^ ^,^ ^23^ In this context, previous studies have shown that sofosbuvir^24^ and simeprevir have promising antiviral effects *in vitro*.^25^ ^,^ ^26^ Recently, we demonstrated the protective effect of sofosbuvir on the vertical transmission of ZIKV and its association with congenital syndrome *in vivo*.^27^ Our new *in vitro* study revealed that simeprevir and sofosbuvir may be repurposed for ZIKV treatment. Additionally, we suggest that the antiviral mechanism enhances NOexpression in host cells.

## MATERIALS AND METHODS

*Protein preparation* - The crystallographic structures of the proteins used for molecular docking in this study were initially obtained from the Protein Data Bank (PDB) (www.rcsb.org). The crystallographic structures acquired were nonstructural protein 3 (NS3 - helicase) of HCV (PDB ID: 3KQN; resolution of 2.05 Å), nonstructural protein 5B (NS5B - RNA polymerase) of HCV (PDB ID: 6MVK; resolution of 2.30 Å; ligand removed), nonstructural protein 3 (NS3 - helicase) of ZIKV (PDB ID: 5JMT; resolution of 1.80 Å), nonstructural protein 5 (NS5 - RNA polymerase) of ZIKV (PDB ID: 5U04; resolution of 1.90 Å), and human iNOS (PDB ID: 4NOS; resolution of 2.25 Å). The proteins were prepared for molecular docking using Discovery Studio Visualizer software (version 2021). Hydrogen charges and Gasteiger charges were added, the water molecules were removed, and the ligands were removed when necessary.

*Ligand preparation* - The molecules sofosbuvir triphosphate (PubChem CID: 90339259), simeprevir (PubChem CID: 24873435), and arginine (PubChem CID: 6322) were obtained from PubChem (pubchem.ncbi.nlm.nih.gov). The structures were collected in SMILES format and inserted into Avogadro software for conformational optimisation. Hydrogen charges were added to all the molecules, and these were prepared for molecular docking.

*Molecular docking* - Molecular docking was performed using AutoDock Vina software (version 1.1.2). Blind docking was used as a parameter to analyse the entire protein structure. The parameters adopted for each structure are as follows: NS3 HCV helicase (centre x = 29.026802; y = -41.463794; z = -20.760335; size x = 40 Å; y = 40 Å; z = 40 Å); NS5B HCV RNA polymerase (centre x = -0.051338; y = 41.178803; z = 19.407154; size x = 45 Å; y = 45 Å; z = 45 Å); NS3 ZIKV helicase (centre x = -12.823207; y = 17.901033; z = -7.910828; size x = 40 Å; y = 40 Å; z = 40 Å); NS5 ZIKV RNA polymerase (centre x = 28.228635; y = 71.679458; z = 103.364635; size x = 31 Å; y = 31 Å; z = 31 Å); and human iNOS (centre x = -1.538500; y = 97.920000; z = 20.469000; size x = 30 Å; y = 30 Å; z = 30 Å).

*Virus assessment* - The ZIKV strain (ES 2916/2015/ID 250) was isolated from the 2015 outbreak in the State of Espírito Santo, Brazil (GenBank: KX197205), with a viral load of 1×10^9^ copies/µL (kindly donated by Dr Ana Bispo from the Laboratory of Flavivirus - IOC - Fiocruz - RJ - Brazil).

*Cell culture* - First, C6/36 cells were used to perform viral titration. The cultivation was maintained with L-15 culture medium supplemented with 10% foetal bovine serum (FBS, Thermo Fisher Scientific, MA, USA) and kept at 28ºC. Subsequently, the C6/36 cell isolate was inoculated into an African green monkey kidney cell line, Vero CCL-81 (Vero CCL-81^TM^, ATCC), which is permissive to flavivirus infection through the expression of the mannose-specific C-type lectins DC-SIGN and L-SIGN, which mediate the entry of ZIKV,^28^ and used for screening drug assays. The cells were maintained in culture medium 199 supplemented with 10% FBS at 37ºC with 5% CO_2_ for seven days.

*Cell treatment* - To confirm the antiviral action of the drugs used in this work on ZIKV infection, we subjected Vero CCL-81 cells to a previous infection with 2×10^7^ copies/µL (MOI = 1). After 1 h, we treated the samples with the compounds at the following concentrations: simeprevir (10 µmol/L), sofosbuvir (10 µmol/L), L-N^G^-nitro arginine methyl ester (L-NAME, iNOS inhibitor) (10 mmol/L), L-norvaline (arginase inhibitor) (10 mmol/L), and dimethyl sulfoxide (DMSO, Sigma-Aldrich, MO, USA) as the compound's diluent (0.05%). The infected and treated cells were subsequently incubated at 37ºC with 5% CO_2_ for seven days before the pellets were collected for viral load evaluation by quantitative real-time polymerase chain reaction (qRT-PCR).

*In vitro evaluation of drug cytotoxicity* - To assess cytotoxicity, we treated the Vero cell line CCL-81 (5x10^5^/well) for 48 h at concentrations four times greater than those used in our protocols: simeprevir and sofosbuvir (40 µmol/L), L-NAME (an iNOS inhibitor) (40 mmol/L), L-norvaline (an arginase inhibitor) (40 mmol/L), and the compound diluent DMSO (0.05%). After this period, the culture supernatants were collected, and the levels of the intracellular enzyme lactate dehydrogenase (LDH) were detected in the medium using a specific kit from Doles (Goiania, Brazil).

*ZIKV replication in Vero cells* - For the qRT-PCR assays, the cells were infected with ZIKV (MOI = 1) and then treated with simeprevir (10 µmol/L), sofosbuvir (10 µmol/L), L-NAME (an iNOS inhibitor) (10 mmol/L), L-norvaline (an arginase inhibitor) (10 mmol/L), or DMSO as the compound's diluent (0.05%). The viral loads in the supernatant and cells were measured by qRT-PCR seven days post-infection. RNA from the culture supernatant was subsequently extracted using an RNeasy Mini Kit and a Qiamp Viral RNA Mini Kit (Qiagen, Hilden, Germany) following the manufacturer's instructions. Intracellular RNA extraction was performed using TRIzol (Sigma-Aldrich, MO, USA) according to the manufacturer's instructions. A one-step qRT-PCR kit, RNAdirect™ Real-time PCR Master Mix (TOYOBO, Japan), and a TaqMan probe were used to quantify the viral RNA produced. The ZIKV primers for qRT-PCR were as follows: sense (TTC GGA ATA TGG AGG CTG AG), antisense (TCG TTT GAG CCT ATC CCA TC), and probe FAM-AGA AAG TGA CCA ACT GGT TGC AGA GCA-BHQ. A standard curve of the serial dilutions was used to quantify the viral RNA yield. All primers were purchased from Invitrogen (Oregon, USA).^29^

*Relative expression of iNOS, ARG-I, and NF-κB by qRT-PCR* - Total RNA was isolated using TRI Reagent (Sigma-Aldrich, MO, USA), and cDNA was synthesised from the RNA using ReadyScript^®^ cDNA Synthesis Mix (Sigma-Aldrich, MO, USA). The following primers were used to analyse ARG-I or iNOS gene expression through qRT-PCR: Arginase-I (ARG-I) primer sense (CTC CAA GCC AAA GTC CTT AGA), antisense (AGG AGC TGC ATT AGG GAC ATC), and iNOS Primer sense (GTT CTC AAG GCA CAG GTC) and antisense (GCA GGT CAC TTA TGT CAC TTA). For NF-κB sense (CGG AAT TCG CCA CCA GAA TGG CAG AAG ATG ATC) and antisense (TGT CAC TCG AGG CAA TTT TGC CTT CTA GAG GTC). All primers were purchased from Invitrogen (Oregon, USA). qRT-PCR was performed by using SYBR green master mix (Sigma-Aldrich, MO, USA) on an ABI 7500 (Applied Biosystems Inc., USA) as described previously.^30^ In qRT-PCR, the comparative CT method (ΔΔCt value) was used to calculate the relative quantification of target genes that were normalised to beta-actin as an internal control using a sense primer (AAG CAG GAG TAT GAC GAG TCC) and an antisense primer (GCC TTC ATA CAT CTC AAG TTG) from Invitrogen (Oregon, USA).^30^

*Nitrite production* - Nitrite production was evaluated in the supernatants, which were obtained through centrifugation (1500 × g/15 min), and 50 µL of each sample was subjected to another treatment with 50 µL of Griess reagent (Sigma-Aldrich, MO, USA). After 30 min, the colorimetric reaction was evaluated, and the absorbance of the chromophore was measured at 540 nm (SpectraMax M5; Molecular Devices, CA, USA). The nitrite concentration was calculated by comparing the final absorbance with the calibration curve. The final absorbance was determined by measuring the absorbance of each sample.^31^

*Data analysis* - The data were analysed using GraphPad Prism software (version 9.3.0 for Windows. GraphPád, CA, USA). Differences between groups were evaluated by one-way analysis of variance (ANOVA), followed by Tukey's post hoc multiple-comparison test. Differences were considered significant when the value was less than 0.05.^32^

## RESULTS

*In silico investigation of the affinity of simeprevir and sofosbuvir for ZIKV proteins* - Simeprevir and sofosbuvir are approved drugs for the treatment of HCV. Our recent data demonstrated that both drugs also exhibit protective effects against ZIKV transmission.^27^ Thus, our initial goal was to investigate whether simeprevir and sofosbuvir could possess a similar mechanism of action against ZIKV. Simeprevir functions as an NS3/4A protease inhibitor, whereas sofosbuvir acts as an NS5B polymerase inhibitor. Both mechanisms disrupt the viral replication process.^24^ ^,^ ^25^ ^,^ ^26^

As shown in Table I, simeprevir demonstrated greater affinity for the NS3 protein of ZIKV than its original target, the HCV helicase (scores of -11.5 kcal/mol and -9.5 kcal/mol, respectively). Only two hydrogen bonds were formed at residues Lys244 and Asp421 for the HCV helicase (Fig. 1A). In contrast, three conventional hydrogen bonds were formed at residues Lys389, His484, and Val543, along with three carbon-hydrogen bonds at Pro432, His486, and Pro542 (Fig. 1B). Carbon-hydrogen bonds are very stable because of the high electronegativity of carbon. Compared with other intermolecular bonds, such as dipole-dipole and van der Waals bonds, carbon-hydrogen bonds are relatively strong. With respect to the ZIKV helicase, simeprevir formed 16 van der Waals interactions, whereas with respect to the HCV helicase, only 10 van der Waals interactions were formed (Fig. 1A-B).

**TABLE I:** Affinity of simeprevir and sofosbuvir against chronic hepatitis C virus (HCV) and Zika virus (ZIKV) proteins *in silico*

Virus	Protein	Ligand	Affinity score (kcal/mol)
HCV	NS3 (helicase)	Simeprevir	-9.5
ZIKV	NS3 (helicase)	Simeprevir	-11.5
HCV	NS5B (RNA polymerase)	Sofosbuvir	-7.1
ZIKV	NS5 (RNA polymerase)	Sofosbuvir	-9.0

**Fig. 1:**
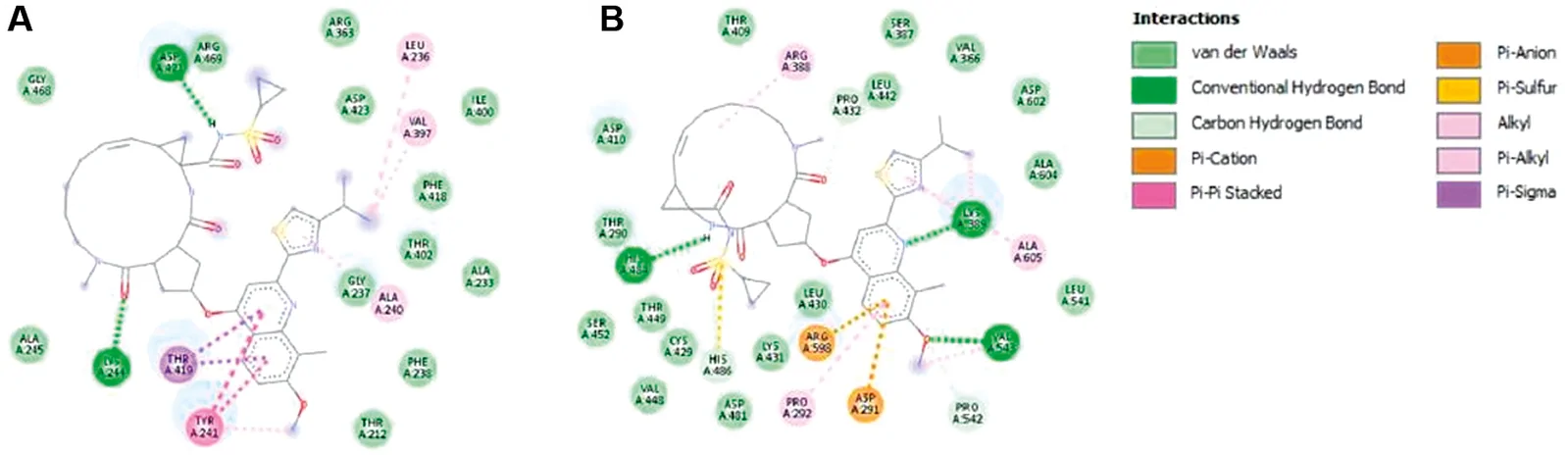
predicted *in silico* interactions of simeprevir with the active site of the helicase (NS3) from chronic hepatitis C virus (HCV) (PDB ID: 3KQN) (A) and Zika virus (ZIKV) (PDB ID: 5JMT) (B).

We also investigated the affinity of sofosbuvir for the NS5 proteins of HCV and ZIKV. Like simeprevir, sofosbuvir demonstrated a greater affinity for the ZIKV protein (Table I). Interestingly, the analysis of interactions revealed that these interactions occurred mainly in the phosphate portion, with a predominance of attractive charges and hydrogen bonds (Fig. 2A-B).

**Fig. 2:**
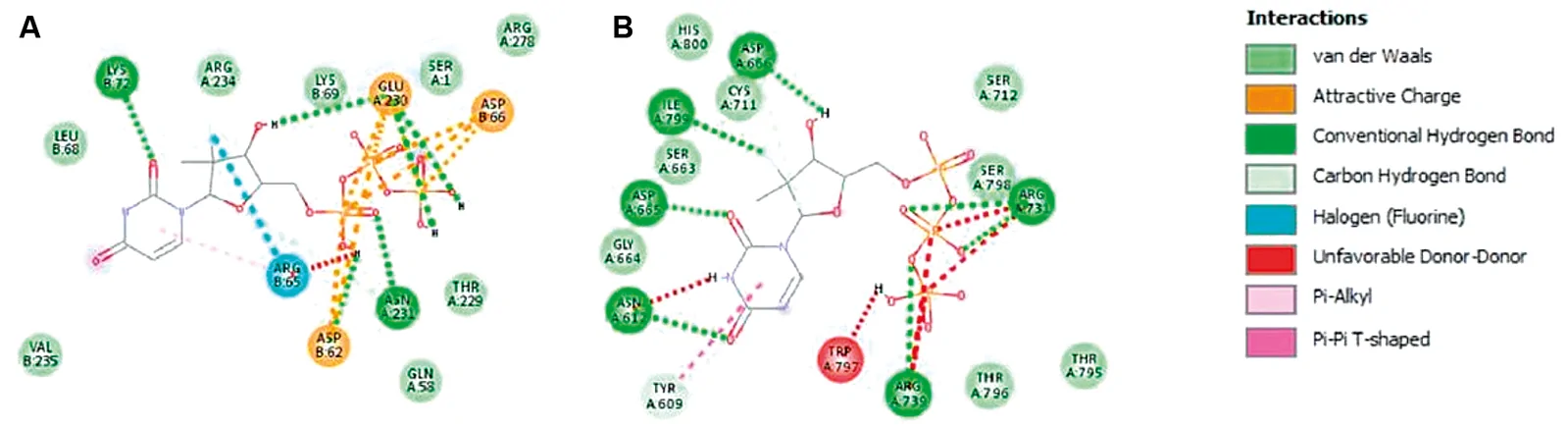
predicted *in silico* interaction of sofosbuvir with the active site of NS5B from chronic hepatitis C virus (HCV) (PDB ID: 6MVK) (A) and NS5 from Zika virus (ZIKV) (PDB ID: 5U04) (B).

*In silico evaluation of the role of simeprevir and sofosbuvir in iNOS inhibition* - Recent investigations have explored the potential effects of simeprevir and sofosbuvir on host inflammatory pathways, particularly iNOS, a critical enzyme involved in immune modulation and oxidative stress.^18^ While iNOS inhibition is not a primary pharmacological mechanism of either agent, emerging evidence suggests that simeprevir may influence iNOS expression indirectly, likely through the modulation of cellular stress responses.^26^ Based on these observations, we evaluated the ability of simeprevir and sofosbuvir to act as potential iNOS inhibitors, aiming to elucidate their possible immunomodulatory effects beyond their antiviral activity.

Both simeprevir and sofosbuvir demonstrated affinity for the human iNOS enzyme, with docking scores of -9.7 and -8.4 kcal/mol, respectively, as presented in Table II. As illustrated in Fig. 3A, simeprevir forms a hydrogen bond with the Thr121 and Arg381 residues. Arg381 also forms two π-cation interactions with the aromatic rings of the ligand. On the other hand, Arg388 forms a π-alkyl interaction, indicating a hydrophobic contact with the aromatic region of the ligand. The interactions between sofosbuvir and iNOS are shown in Fig. 3B. The drug forms hydrogen bonds with residues Arg266, Glu377, Arg382, and Arg388. Like simeprevir, sofosbuvir also presented a π-cation interaction with Arg388. The residues Pro350 and Val352 are involved in hydrophobic interactions (alkyl/π-alkyl) with aromatic or aliphatic parts of the ligand.

**TABLE II:** Affinity of simeprevir and sofosbuvir for inducible nitric oxide synthase (iNOS) protein *in silico*

Protein	Ligand	Affinity score (kcal/mol)
Human iNOS	Simeprevir	-9.7
	Sofosbuvir	-8.4

**Fig. 3:**
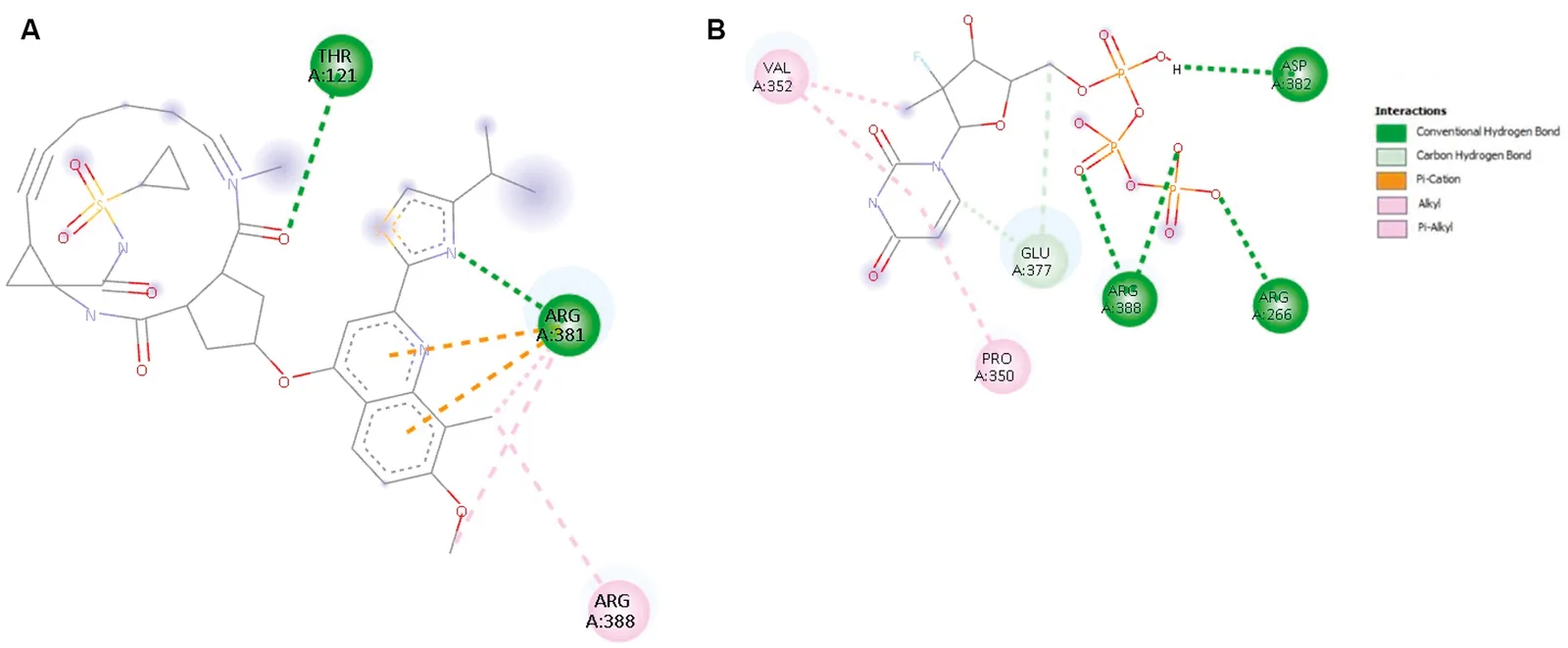
predicted *in silico* interactions of simeprevir (A) and sofosbuvir (B) with the active site of human inducible nitric oxide synthase (iNOS) (PDB ID: 4NOS).

*In vitro evaluation of the ability of simeprevir and sofosbuvir to inhibit ZIKV replication* - After the results indicated that both simeprevir and sofosbuvir have an affinity for ZIKV proteins through a repositioning approach, we investigated whether they would have antiviral effects *in vitro*. In these assays, we used two compounds as controls: L-NAME and L-norvaline. L-NAME is a competitive antagonist of iNOS, thus reducing the availability of NO and serving as a positive control for viral replication.^22^ L-norvaline is a nonselective inhibitor of arginase that increases NO bioavailability by increasing the endogenous stock of L-arginine,^21^ effectively reducing ARG-I expression *in vivo*,^23^ and acts as a negative control for virus replication.

First, we evaluated the possible cytotoxicity of these drugs in the Vero CCL-81 cell line after 48 h of treatment. As demonstrated in Fig. 4A, neither drug induced cell death. We subsequently evaluated viral replication after treatment with these drugs. DMSO and L-NAME did not reduce viral replication compared with that in the infected group (Fig. 4B). In contrast, L-norvaline, simeprevir, and sofosbuvir significantly reduced viral replication by 99% (p < 0.0001), 99% (p < 0.0001), and 98% (p < 0.0001), respectively (Fig. 4B).

**Fig. 4:**
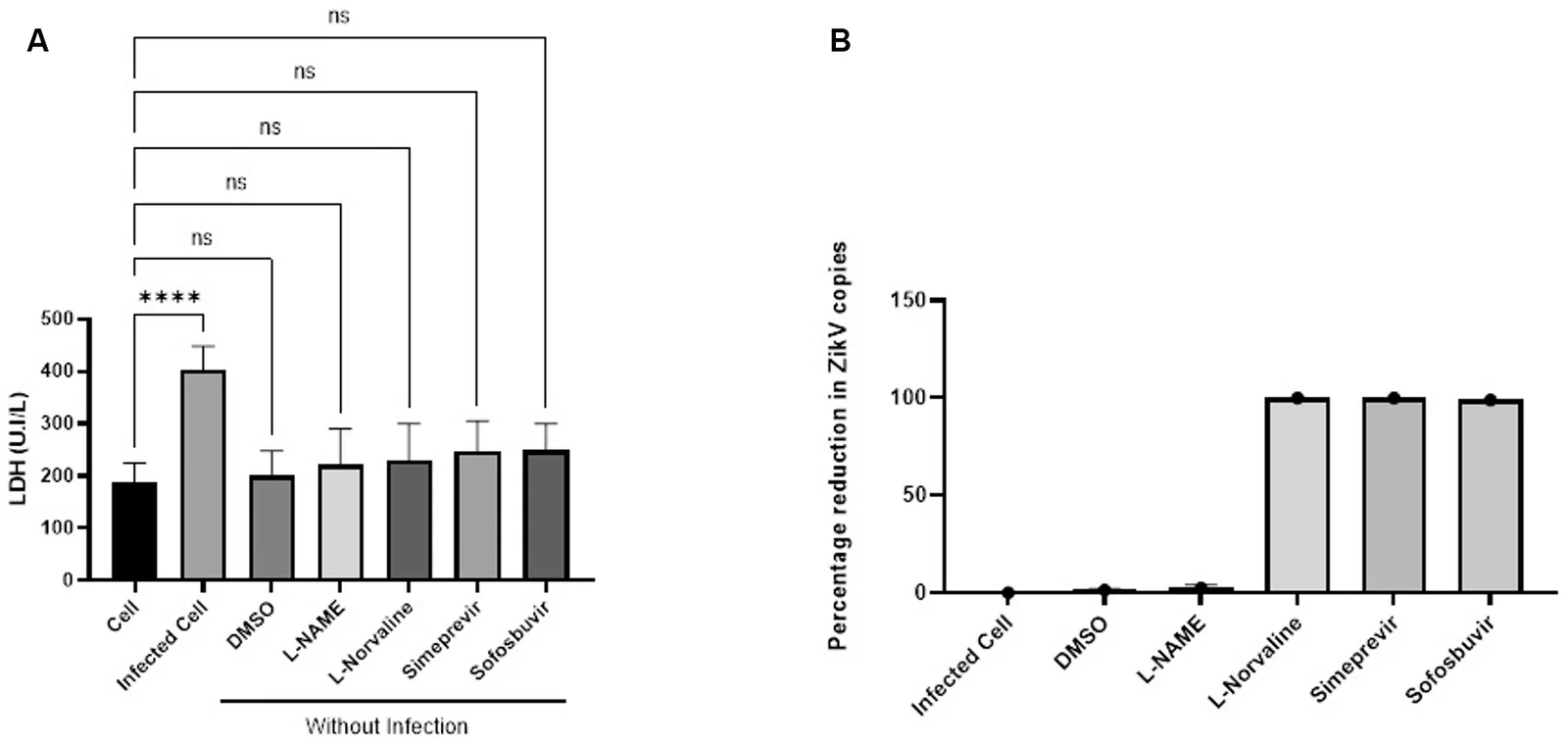
*in vitro* evaluation of simeprevir and sofosbuvir cytotoxicity and Zika virus (ZIKV) replication in Vero CCL-81 cells. (A) The cytotoxicity of simeprevir and sofosbuvir was assessed by quantifying lactate dehydrogenase (LDH) in the supernatant obtained from cells treated for 48 h with drug concentrations four times greater than those used in our protocols: simeprevir and sofosbuvir (40 µmol/L), L-NAME (40 mmol/L), L-norvaline (arginase inhibitor) (40 mmol/L), and the compound diluent dimethyl sulfoxide (DMSO) (0.05%). Infected cells were used as a control for LDH release. (B) Percentage reduction in the number of ZIKV copies in infected Vero CCL-81 cells. Vero CCL-81 cells were infected with ZIKV (2x10⁷ RNA copies/µL) and treated with simeprevir and sofosbuvir (10 µmol/L) and the control compounds L-NAME (10 mmol/L), L-norvaline (10 mmol/L), and the compound diluent DMSO (0.05%). We evaluated the percentage reduction in ZIKV replication after the treatments. The data are shown as the mean ± SDM of three independent experiments (n = 6-9 replicates). P values of 0.05 or less were considered significant, as indicated by the asterisks. ns indicates not significant.

*Simeprevir and sofosbuvir modulate the iNOS pathway in ZIKV-infected cells* - Upon observing that simeprevir and sofosbuvir were capable of inhibiting viral replication, we explored other potential signalling pathways that these drugs might modulate to help host cells combat infection. Since ZIKV infection induces inflammasome activation and increases iNOS levels,^16^ ^,^ ^17^ we investigated how these drugs influence the expression of these proteins. Treatment of Vero CCL-81 cells with simeprevir and sofosbuvir induced increases in iNOS and NF-κB expression (Fig. 5A-B) (p < 0.0001 and p < 0.005, respectively) but not ARG-I expression (Fig. 5C). Once we reported an increase in iNOS expression, we investigated whether the expression of nitrite, a product of NO, was increased in infected cells treated with simeprevir and sofosbuvir. As demonstrated in Fig. 5D, both drugs increased nitrite production in infected cells, similar to L-norvaline, while L-NAME reduced it. Collectively, these data suggest that simeprevir and sofosbuvir inhibit ZIKV replication and increase proinflammatory defences in host cells, aiding in the fight against infection.

**Fig. 5:**
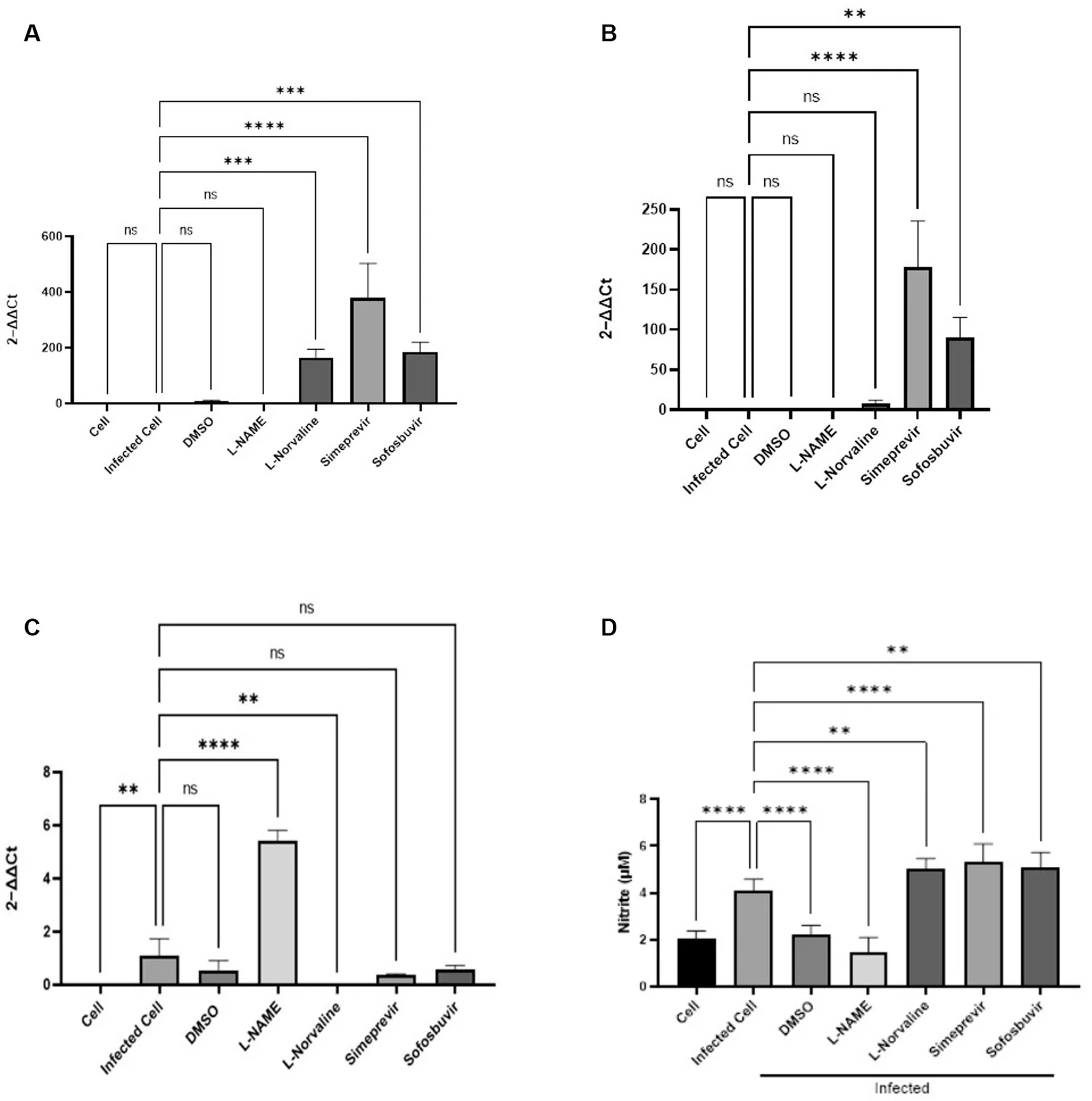
*in vitro* evaluation of the protein expression of simeprevir and sofosbuvir in Zika virus (ZIKV)-infected cells. Quantitative content of inducible nitric oxide synthase (iNOS) (A), NF-κB (B), and ARG-I (C) mRNA by quantitative real-time polymerase chain reaction (qRT-PCR) in Vero CCL-81 cells infected with ZIKV (2x10^7^ RNA copies/µL) and/or treated with L-NAME and L-norvaline (10 mmol/L), simeprevir and sofosbuvir (10 µmol/L), and dimethyl sulfoxide (DMSO) (0.05%). The data are shown as the mean ± SDM of three independent experiments (n = 3). P values of 0.05 or less were considered significant, as indicated by the asterisks. ns indicates not significant. (D) Vero CCL-81 cells were infected with ZIKV (2x10^7^ RNA copies/µL) and/or treated with L-NAME and L-norvaline (10 mmol/L), simeprevir and sofosbuvir (10 µmol/L), or DMSO (0.05%). Cell supernatants were collected to measure nitrite production through the Griess method. The data are shown as the mean ± SDM of three independent experiments (n = 9). P values of 0.05 or less were considered significant, as indicated by the asterisks.

## DISCUSSION

Docking studies targeting ZIKV have focused predominantly on the NS2B-NS3 and NS5 proteins, given their essential roles in viral replication and as promising druggable targets.^33^ ^,^ ^34^ ^,^ ^35^ In this context, our work underscores the repurposing potential of sofosbuvir and simeprevir, revealing interactions with the viral proteins NS5 and NS3, as well as iNOS residues, thereby simultaneously targeting viral replication machinery and host pathways to broaden therapeutic strategies against ZIKV. These antiviral choices were justified by the close relationship between ZIKV and HCV, both members of the Flaviviridae family, with RNA genomes encoding RNA-dependent RNA polymerases that lack molecular proofreading mechanisms.^36^ ^,^ ^37^

In a study conducted by Ejeh et al., simeprevir was reported to interact with the NS3/4A protease/helicase of HCV (PDB ID: 4A92) at residues such as Thr295, Ala413, Thr433, Gln434, Asp454, Val456, Ser489, Val490, Glu493, and Asn556.^38^ Additionally, Badshah et al. reported that the NS3 helicase of ZIKV (PDB ID: 5JRZ) interacts with benzothiazine analogues, proposed as potential inhibitors of ZIKV, with molecules binding mainly to residues Lys200, Thr201, Arg459, and Arg462.^39^ In comparison, our results revealed binding residues that are located near those reported in these studies. Notably, the proteins investigated differed; nevertheless, the proximity of these sites suggests overlapping binding regions across HCV and ZIKV.

Sofosbuvir has been shown to interact with ZIKV NS5B polymerase at amino acid residues similar to those previously reported, including Thr795, Thr796, Trp797, Ser798, and Ile799.^40^ ^,^ ^41^ Sofosbuvir also binds to one of the key catalytic residues, Asp665, which, together with Asp535, corresponds to the active site of ZIKV NS5 RNA-dependent RNA polymerase (RdRp). Importantly, these catalytic interactions converge with structural motifs essential for polymerase function. Among them, the priming loop (residues Val785-Asp810) plays a critical role by allosterically positioning the 3′ terminus of the RNA template at the active site.^42^ The interaction of sofosbuvir with both catalytic residues (Asp665 and Asp535) and the priming loop (Val785-Asp810) demonstrates its ability to target multiple functional regions of ZIKV NS5 RdRp, supporting its potential as a repurposed antiviral candidate.

Sofosbuvir interacted with the principal substrate-binding residues of iNOS, namely, Glu377 and Asp382, among other nearby residues.^43^ These interactions suggest that repurposing sofosbuvir or designing larger competitive inhibitors inspired by its scaffold could provide a promising strategy for improved iNOS inhibition.

NO is an endogenous multifunctional molecule with key antimicrobial and anti-inflammatory effects on pulmonary vascular function in the context of viral infection.^44^ NO is also involved in the pathogenesis of many viral infections, as well as in direct or indirect antiviral activities, such as directly inactivating viral particles, blocking viral replication, and modulating the host immune response.^44^

NO production is a known interferon (IFN) type I and II antiviral mechanism through the stimulation of iNOS, which was recently reviewed.^45^ In terms of host innate immunity, iNOS production may also be observed *in vivo*; it is strongly expressed in liver samples of acute hepatitis A virus (HAV) from *Callithrix jacchus* challenged with HAV strain HAF-203, with injured hepatocytes releasing their intracellular liver enzymes (alanine aminotransferase (ALT) and aspartate aminotransferase (AST)) to the bloodstream.^46^

In the experimental part of this study, the choice of epithelial Vero CCl-81 cell line is justified by its susceptibility to ZIKV infection, efficient viral replication, and clear cytopathic effects.^28^ ^,^ ^47^ ^,^ ^48^ Additionally, nitric oxide production *in vitro* has been previously reported.^49^

LDH levels in the supernatant of the CCL81 Vero cell cultures were used to exclude the possibility of drug-induced cytotoxicity by the two repurposed antivirals clinically approved for HCV treatments, simeprevir and sofosbuvir.^50^ No drug-related toxicity was detected even when cells were exposed to concentrations four times higher than those established in 48-h antiviral protocols, confirming the absence of cytotoxicity. The observed increase in LDH in the supernatant of ZIKV-infected Vero CCl-81 cells was instead attributable to the cytopathic effect induced by ZIKV infection,^47^ supporting the use of LDH as a reliable marker of Vero CCL-81 cell injury and in contrast with previous reports.^51^

With respect to antiviral activity, the concentration of nitrite released into the supernatant of ZIKV-infected Vero CCL-81 cultures confirmed the increase in NO levels induced by ZIKV infection. In parallel, treatment with DAAs significantly reduced ZIKV RNA copy numbers in the supernatants, with simeprevir and sofosbuvir both having marked inhibitory effects.

L-norvaline, an L-arginase inhibitor that enhances NO biosynthesis, produced a similar reduction in viral RNA content to that observed with DAAs. Consistent with previous reports, L-norvaline treatment of Vero E6, A549-ACE2, and Calu-3 cells induced NO production and inhibited SARS-CoV-2 replication.^52^ In Vero cells, intracellular ARG-I activity is competitively inhibited by L-arginine and L-norvaline,^53^ leading to increased intracellular L-arginine availability for iNOS activity, elevated NO production, and increased iNOS mRNA expression.^54^ Similarly, L-norvaline, simeprevir, and sofosbuvir increased the intracellular NO concentration, consequently reducing ZIKV replication in Vero CCl-81 cells and suggesting an additional antiviral mechanism of action for both DAAs. According to the literature, the classical inhibitory effect of the active metabolite of sofosbuvir is mediated through inhibition of ZIKV RNA polymerase activity,^24^ ^,^ ^25^ ^,^ ^55^ whereas simeprevir was originally classified as an HCV NS3/4A protease inhibitor.^56^ Importantly, the Vero CCl-81 cell line used in this study is capable of metabolising sofosbuvir into its active form, as supported by the antiviral activity demonstrated.^57^ ^,^ ^58^

In the group of drugs that increase intracellular NO levels (L-norvaline, simeprevir, and sofosbuvir), distinct effects on ARG-I transcription were observed. L-NAME increased ARG-I expression, whereas L-norvaline inhibited ARG-I expression, and DAAs did not significantly influence ARG-I expression. These findings suggest that the mechanisms leading to elevated intracellular NO production are closely dependent on L-arginine availability and ARG-I activity. L-norvaline regulates the transcription of IFN-β and ISG-15, as well as other innate immune pathways, which are also activated by simeprevir.^26^ However, the classic antiviral mechanism of simeprevir is mediated primarily through inhibition of the ZIKV NS2B/NS3 protease,^25^ whereas the active metabolite of sofosbuvir targets the enzymatic function of the viral RdRp domain.^24^

We also examined the relative expression of NF-ĸB, a transcription factor that regulates diverse biological processes, including the induction of the iNOS gene.^59^ Interestingly, ZIKV-infected cells did not exhibit increased NF-kB mRNA transcription, in contrast to the upregulation observed in cultures treated with L-NAME, L-norvaline, or both DAAs. These findings differ from those of previous reports in which HAV infection enhanced NF-κB transcription independently of the PKR-induced pathway.^60^

Previous studies have shown that iNOS activity depends on a continuous supply of L-arginine from the extracellular compartment, with L-arginine transport via CAT-2B being essential for increased NO production associated with increased iNOS expression in heart failure.^54^ Consistently, L-arginine levels are closely linked to iNOS mRNA transcription. L-NAME, an L-arginine analogue, reduced the supernatant NO content by approximately 60% through inhibition of iNOS activity but did not affect ZIKV RNA replication in the Vero CCl-81 cell line. The amount of ZIKV RNA in the supernatant remained comparable to that in the untreated controls (infected cells and DMSO). In contrast, L-NAME treatment significantly increased ARG-I mRNA transcription in Vero CCl-81 cells, likely because L-NAME inhibits iNOS activity without affecting L-arginine transport.^54^ Moreover, elevated intracellular L-arginine levels serve as a substrate that induces ARG-I transcription in ZIVK-infected Vero CCl-81 cells, thereby increasing catalytic activity.^23^

Taken together, our findings highlight an additional mechanism of action for these two DAAs, which is mediated through activation of the NO pathway during ZIKV infection. This host-targeted effect complements their classical antiviral activities, with sofosbuvir already demonstrating promising *in silico* and *in vitro* antiviral activity.^61^ These results also underscore the relevance of L-arginine metabolism and iNOS regulation in shaping the cellular response to infection. Although iNOS inhibitors have been proposed as neuroprotective agents during pregnancy because of their ability to mitigate severe inflammatory responses in the developing brain,^62^ ^,^ ^63^ further studies are needed to substantiate this evidence. Collectively, these insights provide new perspectives for therapeutic intervention and support the development of efficient and safe treatments against ZIKV infection.

## ACKNOWLEDGEMENTS

To Fundação Oswaldo Cruz for providing structural support and Dr Ana Bispo of the Laboratório de Flavivírus at the Instituto Oswaldo Cruz (IOC) for kindly providing the Zika virus strain.

## Data Availability

The contents underlying the research text are included in the manuscript.
