# Supplementary material for: Enhancement of simeprevir and sofosbuvir anti-ZIKV activity by iNOS-derived NO in vitro
Source: Mem Inst Oswaldo Cruz. 2026 Sep 28;121:e250315. doi: 10.1590/0074-02760250315 (PMC13623229; doi:10.1590/0074-02760250315)
Supplement: Peer review history

# Reviewer report: FIRST REVIEW ROUND - REVIEWERS' COMMENTS

## REVIEWER #1

Dear Editor and Authors,

First of all, I would like to thank you for the invitation to review an article on such an important topic as ZIKV infection in such a prestigious journal as MOC. I believe the article is relevant, but certain aspects should be verified before proceeding with publication.

a) Adequacy of the title/abstract/introduction;

• The title could be clearer in referring to internally produced NO. The first time I read it, it seemed to be an added NO, and that struck me as odd. I suggest being more specific in the title of the work and modifying it to something like:

"iNOS-Mediated Nitric Oxide Enhances the Anti-ZIKV Activity of Simeprevir and Sofosbuvir in vitro."

Or

"Enhancement of Simeprevir and Sofosbuvir Anti-ZIKV Activity by iNOS-Derived NO in vitro"

• On page 6, line 25, you could comment that there are phase I and/or II clinical trials for vaccines and treatments for this disease; however, with the reduction in epidemic cases worldwide after 2017, the continuation of these trials was affected.

(Lee HJ, Abu Bakar S, Shin OS. An updated review of Zika virus vaccine development. Clin Exp Vaccine Res. 2025 Oct;14(4):325-334. doi: 10.7774/cevr.2025.14.e42. Epub 2025 Oct 21. PMID: 41221169; PMCID: PMC12599434.)

b) Originality and importance of the contribution for the development of the field of study. And relevance of.

I believe in the importance of this study in clarifying the mechanisms of action of the substances tested in an extremely serious disease, such as that caused by ZIKV. It should be noted that the present study involves trials that may facilitate a potential repositioning of this drug for a disease currently lacking a marketed treatment.

c) Methodology, results, and discussion;

• It would be interesting to see tests with other strains from Brazil, the main country affected by the ZIKV-related epidemic, since the manifestations and clinical responses of viral infections can vary according to the ZIKV strain from that time.

• It's important to check the state of the art. Are there really no other articles with the same research? In vitro or in silico?

d) References;

Try to find more references than 16 and 17 concerning the subject mentioned above in the second dot at Topic C of this revision.

e) Figures and tables.

• In the captions, specify whether the activities are in vitro or in silico.

• Honestly, I believe I haven't found all the figures from the text. For me, only one figure appears with three images: A, B, and "Interactions". The captions and text mention five figures. I don't know if they weren't attached correctly or if it was a misunderstanding on my part, but it's really confusing. It needs a review and a proper explanation.

Best regards!

## AUTHORS' RESPONSE TO THE REVIEWERS

Dear Dr. Bezerra:

Manuscript ID MIOC-2025-0315 entitled "Nitric oxide enhances the anti-ZIKV activity of Simeprevir and Sofosbuvir in vitro" which you submitted to the Memórias do Instituto Oswaldo Cruz, has been fully evaluated by independent peer reviewers and substantial concerns were raised about the manuscript as it currently stands. These issues must be addressed before we would be willing to consider a revised version of your study.

Please be aware that the submission of a new revised version of your manuscript does not guarantee eventual acceptance, being subject to re-review before a decision is rendered.

If you are able to revise your manuscript along the lines recommended by the referees within 90 days, a list of specific responses to each of the reviewer's comments, noting where you have made such changes in the revision, is needed. Authors are not mandated to agree with every suggestion made by the reviewer. However, reasons for contesting any reviewer comments must be provided. If those unresolved comments appear to be fundamental to the work being described, the revision may be resubmitted to the Editors for final decision.

The specific response to the reviewer's comments, the new version of the manuscript, including tables and legends to figures must be submitted. If it is the case, all figures (in TIFF format 300 dpi), must be uploaded again separately with legends. Please upload the new version of manuscript in the "manuscript files" section as main documents.

To revise your manuscript, log into https://mc04.manuscriptcentral.com/mioc-scielo and enter your Author Center, where you will find your manuscript under "Manuscripts with Decisions." Under "Actions," click on "Create a Revision." Your manuscript number has been appended to denote a revision.

You may also click the link below to start the revision process for your manuscript. If you use the link below you will not be required to login to ScholarOne Manuscripts.

* PLEASE NOTE: This is a two-step process. After clicking on the link, you will be directed to a webpage to confirm.* https://mc04.manuscriptcentral.com/mioc-scielo?URL_MASK=483ec79f798f4ae9bce762230169845c

You will be unable to make your revisions on the originally submitted version of the manuscript. Instead, revise your manuscript using a word processing program and save it on your computer. Please also highlight the changes to your manuscript within the document by using the track changes mode in MS Word or by using bold or colored text.

Once the revised manuscript is ready, you can upload and submit through your Author Center.

When submitting your revised manuscript, you will be able to respond to the comments made by the reviewer(s) in the space provided. You can use this space to document any changes you make to the original manuscript. In order to expedite the processing of the revised manuscript, please be as specific as possible in your response to the reviewer(s).

IMPORTANT: Your original files are available to you when you upload your revised manuscript. Please delete any redundant files before completing the submission.

Once again, thank you for submitting your manuscript to the Memórias do Instituto Oswaldo Cruz and I look forward to receiving your revision.

Sincerely,

Dr. Adeilton Brandão

Handling Editor

Memórias do Instituto Oswaldo Cruz

Rio de Janeiro, April 14, 2026.

Dear Dr. Adeilton Brandão,

Please find enclosed the revised manuscript entitled "Nitric oxide enhances the anti-ZIKV activity of Simeprevir and Sofosbuvir *in vitro*". We have taken all reviewer comments into consideration, as displayed below in a point-by-point response to each issue and/or comment. Major modifications are labeled in red in the text. We hope that the manuscript is now publishable in Memórias do Instituto Oswaldo Cruz. We look forward to hearing from you as soon as possible.

Sincerely,

Dr. Rômulo José Soares Bezerra

Laboratory for Technological Development in Virology

Oswaldo Cruz Institute

Rio de Janeiro - Brazil

**REVIEWER COMMENTS:**

**Reviewer: 1**

Dear Editor and Authors,

First of all, I would like to thank you for the invitation to review an article on such an important topic as ZIKV infection in such a prestigious journal as MOC. I believe the article is relevant, but certain aspects should be verified before proceeding with publication.

**a) Adequacy of the title/abstract/introduction;**

* The title could be clearer in referring to internally produced NO. The first time I read it, it seemed to be an added NO, and that struck me as odd. I suggest being more specific in the title of the work and modifying it to something like: "iNOS-Mediated Nitric Oxide Enhances the Anti-ZIKV Activity of Simeprevir and Sofosbuvir in vitro." Or "Enhancement of Simeprevir and Sofosbuvir Anti-ZIKV Activity by iNOS-Derived NO in vitro"

*R:* Following your suggestions, we changed the title of the manuscript.

* On page 6, line 25, you could comment that there are phase I and/or II clinical trials for vaccines and treatments for this disease; however, with the reduction in epidemic cases worldwide after 2017, the continuation of these trials was affected.

(Lee HJ, Abu Bakar S, Shin OS. An updated review of Zika virus vaccine development. Clin Exp Vaccine Res. 2025 Oct;14(4):325-334. doi: 10.7774/cevr.2025.14.e42. Epub 2025 Oct 21. PMID: 41221169; PMCID: PMC12599434.)

*R:* Thank you for your suggestion. The manuscript has been revised to incorporate the suggested information and references in the Introduction section.

**b) Originality and importance of the contribution for the development of the field of study. And relevance of.** I believe in the importance of this study in clarifying the mechanisms of action of the substances tested in an extremely serious disease, such as that caused by ZIKV. It should be noted that the present study involves trials that may facilitate a potential repositioning of this drug for a disease currently lacking a marketed treatment.

*R:* We thank you for recognizing the originality, relevance, and potential impact of our study on ZIKV.

**c) Methodology, results, and discussion;**

* It would be interesting to see tests with other strains from Brazil, the main country affected by the ZIKV-related epidemic, since the manifestations and clinical responses of viral infections can vary according to the ZIKV strain from that time.

*R:* Thank you for your suggestion. In this work, we performed a pilot test using a Brazilian ZIKV strain. The preliminary results were consistent with our main findings, supporting the relevance of our observations. We agree that expanding the analysis to include a broader panel of Brazilian strains would provide a more comprehensive understanding, and we plan to continue this line of investigation in future studies.

* It's important to check the state of the art. Are there really no other articles with the same research? In vitro or in silico?

*R:* Thank you for your suggestion. We inserted five new paragraphs in the discussion section (pages 18, 19, and 22, highlighted in red) to address the state of the art, including both *in silico* and *in vitro* studies. However, such reports remain scarce, which underscores the novelty and relevance of our findings.

**d) References;**

Try to find more references than 16 and 17 concerning the subject mentioned above in the second dot at Topic C of this revision.

*R:* Thank you for your suggestion. We inserted 14 new references related to Topic C (references 33--43 and 61--63, highlighted in red) to strengthen the discussion.

**e) Figures and tables.**

* In the captions, specify whether the activities are in vitro or in silico.

* Honestly, I believe I haven't found all the figures from the text. For me, only one figure appears with three images: A, B, and "Interactions". The captions and text mention five figures. I don't know if they weren't attached correctly or if it was a misunderstanding on my part, but it's really confusing. It needs a review and a proper explanation.

*R:* We thank you for your observation. We carefully reviewed all figures and citations throughout the manuscript and corrected them where necessary. In addition, we incorporated the terms "*in silico*" and "*in vitro*" into the captions to enhance clarity.

Best regards!

# Reviewer report: REVIEWERS' COMMENTS

## REVIEWER #1

Dear Editor,

All requested revisions have been successfully implemented. I recommend the manuscript for publication in its current form.

Best wishes.
